# The T160A hemagglutinin substitution affects not only receptor binding property but also transmissibility of H5N1 clade 2.3.4 avian influenza virus in guinea pigs

**DOI:** 10.1186/s13567-017-0410-0

**Published:** 2017-02-06

**Authors:** Min Gu, Qunhui Li, Ruyi Gao, Dongchang He, Yunpeng Xu, Haixu Xu, Lijun Xu, Xiaoquan Wang, Jiao Hu, Xiaowen Liu, Shunlin Hu, Daxin Peng, Xinan Jiao, Xiufan Liu

**Affiliations:** 1 College of Veterinary Medicine, Yangzhou University, Yangzhou, Jiangsu 225009 China; 2 Jiangsu Key Laboratory of Zoonosis, Yangzhou University, Yangzhou, Jiangsu 225009 China; 3 Yangzhou Entry-Exit Inspection and Quarantine Bureau, Yangzhou, Jiangsu 225009 China; 4 Jiangsu Co-Innovation Center for the Prevention and Control of Important Animal Infectious Disease and Zoonoses, Yangzhou, Jiangsu 225009 China

## Abstract

We generated and characterized site-directed HA mutants on the genetic backbone of H5N1 clade 2.3.4 virus preferentially binding to α-2,3 receptors in order to identify the key determinants in hemagglutinin rendering the dual affinity to both α-2,3 (avian-type) and α-2,6 (human-type) linked sialic acid receptors of the current clade 2.3.4.4 H5NX subtype avian influenza reassortants. The results show that the T160A substitution resulted in the loss of a glycosylation site at 158N and led not only to enhanced binding specificity for human-type receptors but also transmissibility among guinea pigs, which could be considered as an important molecular marker for assessing pandemic potential of H5 subtype avian influenza isolates.

**Electronic supplementary material:**

The online version of this article (doi:10.1186/s13567-017-0410-0) contains supplementary material, which is available to authorized users.

## Introduction, methods, and results

The highly pathogenic Asian-lineage avian influenza (HPAI) H5N1 viruses pose a serious pandemic threat due to their high virulence and mortality, and their increasingly expanding host range, as well as the significant ongoing evolution toward efficient transmission in mammalian models [1]. Despite the fact that the HPAI H5N1 viruses have been globally distributed and endemic in several countries, the direct transmission from avian species to human beings still remains a relatively rare event [2]. As acknowledged, the preference of hemagglutinin (HA) of influenza A virus for particular glycan-linkage of terminal sialic acid (SA) moieties on susceptible cells is a key determinant of viral host range and tissue tropism [3]. Generally, avian influenza viruses preferentially bind to SA in α-2,3 linkage with the vicinal galactose (α-2,3 SA), which predominates in the intestinal tracts of waterfowl, the natural reservoir of influenza A viruses. In contrast, human-adapted influenza viruses showed selective binding affinity to SA in α-2,6 pattern (α-2,6 SA), the major linkage type in human respiratory epithelia [4].

According to the literature, some particular amino acid substitutions in the vicinity of the HA receptor-binding domain (RBD) that contributed to the adaptation of avian influenza viruses to human hosts have been elucidated for the previous influenza pandemic subtypes, even if just one or two critical residues could be responsible for host switching since the RBD pocket is shallow. For instance, the E190D (H3 numbering throughout the paper) and G225D/E mutations in HA of the H1N1/1918 pandemic viruses, as well as the Q226L and G228S substitutions in HA of the H2N2/1957 and H3N2/1968 pandemic viruses, correlated with the conversion of binding preference to SA receptors from α-2,3 to α-2,6 type [3, 5–7]. However, most HPAI H5N1 viruses do not bind to α-2,6 receptors with high affinity [8, 9], although the number of cumulative human cases available has already reached 854 with over 50% mortality until July, 2016. And it is widely believed that the restriction of receptor binding specificity is the major factor preventing the H5N1 virus from efficiently transmitting from person to person and causing a pandemic [10].

In the last few years, natural reassortant HPAI H5NX viruses with various NA subtypes other than N1, at least including N2, N6 and N8, in the newly designated clade 2.3.4.4 affiliating to clade 2.3.4 have been circulating in many areas in China [11, 12]. In addition, the novel H5N8 virus has invaded multiple countries across Asia, Europe and North-America mainly via migratory birds, leading to further subsequent reassortment with local lineages of influenza virus in wild birds to generate devastating variants like the HPAI H5N2 [11, 13]. Moreover, the H5N6 clade 2.3.4.4 expanding to Laos and Viet Nam gained ecological niches there and not only resulted in losses to the poultry industry but also caused human death [14–16].

Noteworthy, as compared with the H5N1 viruses in clade 2.3.4 showing complete α-2,3 receptor specificity, most of those above mentioned HPAI H5NX reassortant viruses can bind to both α-2,3 (avian-type) and α-2,6 (human-type) linked sialic acid receptors [17–22]. However, the molecular basis conferring this dual receptor binding property has not been clearly described yet. Therefore in this study, we generated a series of site-directed HA mutants based on the genetic backbone of H5N1 clade 2.3.4 virus, to determine the key amino acid contributing to the increased viral affinity to α-2,6 receptors and the effect on viral transmissibility among guinea pigs. All experiments involving live viruses were conducted in animal biosafety level 3 facilities in Yangzhou University. All the animal studies were permitted by the Department of Science and Technology of the Jiangsu Province (license ID: SYXK (SU) 2016-0019), and complied with the guidelines of the institutional administrative committee and ethics committee of laboratory animals.

Firstly, the HA sequences of the previously reported H5NX clade 2.3.4.4 reassortants possessing dual receptor binding property were retrieved from the GenBank and GISAID public databases to be aligned with that of H5N1 clade 2.3.4 virus A/mallard/Huadong/S/2005 (HD/05) exhibiting just α-2,3 receptor specificity through the software MEGA (version 6.06). As shown in Table 1, six consistent amino acid substitutions all located in the HA1 subunit including K90R, T160A, K222Q, S227R, N244H and A266T were identified (Additional file 1). In particular, we noticed that the T to A mutation at site 160 simultaneously resulted in the loss of the glycosylation site at position 158N of the HA protein.

**Table 1:** HA amino acid variations between A/mallard/Huadong/S/2005(H5N1) and the H5NX clade 2.3.4.4 reassortant viruses

Virus	HA sequence ID in public database	Reference	Clade	Receptor binding specificity	Amino acid variation in HA^a^					
					90	160	222	227	244	266
A/mallard/Huadong/S/2005(H5N1)	EU195392^b^	[17]	2.3.4	α-2,3	K	T	K	S	N	A
A/duck/Jiangsu/k1203/2010(H5N8)	JQ973694^b^	[17]	2.3.4.4^d^	α-2,3+α-2,6	***R*** ^e^	***A***	***Q***	***R***	***H***	***T***
A/duck/Shandong/Q1/2013(H5N8)	KM504101^b^	[17]	2.3.4.4^d^	α-2,3+α-2,6	***R*** ^e^	***A***	***Q***	***R***	***H***	***T***
A/duck/Eastern China/1111/2011(H5N2)	JQ041401^b^	[17]	2.3.4.4^d^	α-2,3+α-2,6	***R*** ^e^	***A***	***Q***	***R***	***H***	***T***
A/goose/Eastern China/1112/2011(H5N2)	JQ041402^b^	[17]	2.3.4.4^d^	α-2,3+α-2,6	***R*** ^e^	***A***	***Q***	***R***	***H***	***T***
A/duck/Eastern China/008/2008(H5N5)	GU727653^b^	[18]	2.3.4.4	α-2,3+α-2,6	***R*** ^e^	***A***	***Q***	***R***	***H***	***T***
A/duck/Eastern China/031/2009(H5N5)	GU727661^b^	[18]	2.3.4.4	α-2,3+α-2,6	***R*** ^e^	***A***	***Q***	***R***	***H***	***T***
A/goose/Shandong/k1204/2009(H5N5)	JQ973670^b^	[18]	2.3.4.4	α-2,3+α-2,6	***R*** ^e^	***A***	***Q***	***R***	***H***	***T***
A/goose/Guangdong/k0103/2010(H5N5)	JQ973686^b^	[18]	2.3.4.4	α-2,3+α-2,6	***R*** ^e^	***A***	***Q***	***R***	***H***	***T***
A/quail/Jiangsu/k0104/2010(H5N5)	JQ973678^b^	[18]	2.3.4.4	α-2,3+α-2,6	***R*** ^e^	***A***	***Q***	***R***	***H***	***T***
A/goose/Jiangsu/QD5/2014(H5N8)	KT221066^b^	[19]	2.3.4.4	α-2,3+α-2,6	***R*** ^e^	***A***	***Q***	***R***	***H***	***T***
A/goose/Shandong/WFSG1/2014(H5N8)	KT221074^b^	[19]	2.3.4.4	α-2,3+α-2,6	***R*** ^e^	***A***	***Q***	***R***	***H***	***T***
A/goose/Yangzhou/0420/2014(H5N8)	KT221082^b^	[19]	2.3.4.4	α-2,3+α-2,6	***R*** ^e^	***A***	***Q***	***R***	***H***	***T***
A/goose/Eastern China/CZ/2013(H5N8)	KX013029^b^	[20]	2.3.4.4	α-2,3+α-2,6	***R*** ^e^	***A***	***Q***	***R***	***H***	***T***
A/duck/Eastern China/JY/2014(H5N8)	KX013021^b^	[20]	2.3.4.4	α-2,3+α-2,6	***R*** ^e^	***A***	***Q***	***R***	***H***	***T***
A/goose/Eastern China/S0513/2013(H5N6)	KP732638^b^	[21]	2.3.4.4	α-2,3+α-2,6	***R*** ^e^	***A***	***Q***	***R***	***H***	***T***
A/duck/Eastern China/S0711/2014(H5N6)	KP732641^b^	[21]	2.3.4.4	α-2,3+α-2,6	***R*** ^e^	***A***	***Q***	***R***	***H***	***T***
A/duck/Eastern China/S0908/2014(H5N6)	KP732643^b^	[21]	2.3.4.4	α-2,3+α-2,6	***R*** ^e^	***A***	***Q***	***R***	***H***	***T***
A/goose/Eastern China/S0322/2014(H5N6)	KP732644^b^	[21]	2.3.4.4	α-2,3+α-2,6	***R*** ^e^	***A***	***Q***	***R***	***H***	***T***
A/migratory waterfowl/Hubei/Chenhu1347/2014(H5N6)	EPI_ISL_234377^c^	[22]	2.3.4.4	α-2,3+α-2,6	***R*** ^e^	***A***	***Q***	***R***	***H***	***T***
A/Anascrecca/Hubei/Chenhu1623-5/2014(H5N6)	EPI_ISL_179644^c^	[22]	2.3.4.4	α-2,3+α-2,6	***R*** ^e^	***A***	***Q***	***R***	***H***	***T***

^a^H3 numbering, amino acids were in single-letter abbreviation. ‘‘K’’ is lysine, ‘‘R’’ isargnine, ‘‘S’’ isserine, ‘‘T’’ is threonine, ‘‘A’’ is alanine, ‘‘Q’’ is glutamine, ‘‘N’’ is asparagine, and ‘‘H’’ is histidine.

^b^HA sequence ID in GenBank.

^c^HA sequence ID in GISAID.

^d^Although the H5 subtype virus was initially clustered into clade 2.3.4.6 when published in Ref. [17], the provisional clade 2.3.4.6 designation has already been replaced by the unified classification of clade 2.3.4.4.

^e^HA amino acid variations from the H5N1 clade 2.3.4 viruses are indicated in bolditalic.

Subsequently, we introduced each of these six substitutions individually into the HA of HD/05 virus by a site-directed mutagenesis kit (TransGen, Beijing, China) to generate site-specific HA mutants via reverse genetics. Briefly, a mixture of 293T and MDCK cells was transfected with PHW2000-vectored plasmids encoding all eight influenza A virus genes using the PolyFect transfection reagent (Qiagen, Valencia, CA, USA) as recommended by the manufacturer. After 48 h, the supernatant was harvested and inoculated into 10-day-old SPF embryonated chicken eggs for propagation of stock virus. All of the rescued viruses were confirmed by sequencing of their viral RNA, and no other amino acid changes in the viral genome apart from the expected mutated site in HA genes were observed, even when the rescued viruses were passaged for 5 generations in eggs.

To evaluate the receptor binding property of the six rescued HA mutants, we conducted the solid-phase direct binding assay with corresponding α-2,3 and α-2,6 linked glycans as previously described [17]. In short, 96-well microtiter plates were coated with the appropriate concentration of synthetic sialylglycopolymers Neu5Aca2-3Galb1-4GlcNAcb (3′SLN)-PAA-biotin and Neu5Aca2-3Galb1-4GlcNAcb (6′SLN)-PAA-biotin (GlycoTech, Rockville, MD, USA) at 4 °C overnight and blocked with PBS containing 2% skim milk powder before incubating with tested live virus in triplicate. Then, specific primary antibodies and HRP-conjugated anti-IgG secondary antibodies were added sequentially into the well to react with the virus. Finally, tetramethylbenzidine was used as a chromogenic substrate by terminating the reaction with 1 M H_2_SO_4_, and the absorbance at 450 nm was read for curve plotting.

**Figure 1:**
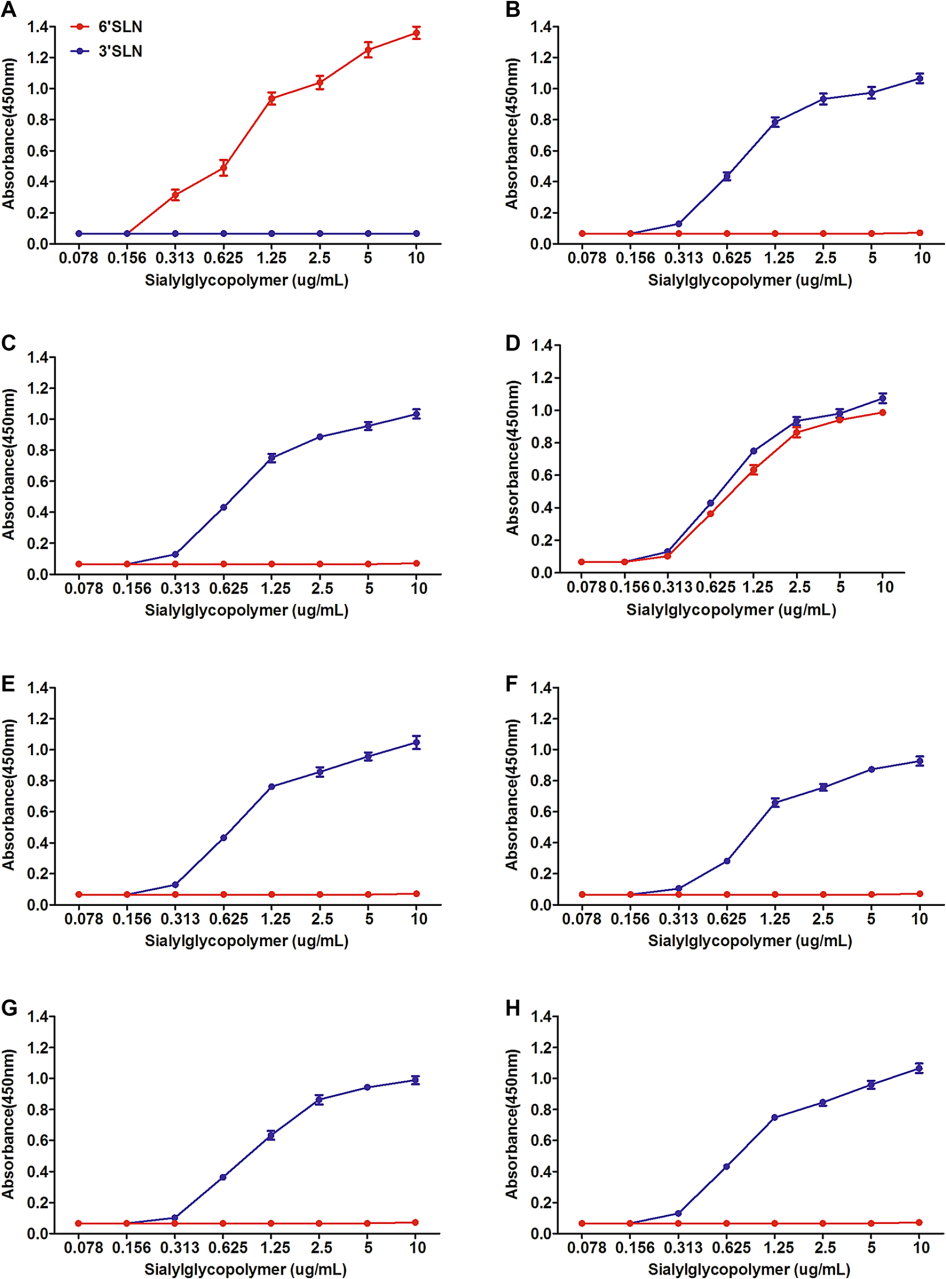
**Solid-phase receptor-binding assay of HA mutants based on HD/05 virus.** Solid-phase receptor-binding assay of human isolate CA/09 (**A**), poultry isolate HD/05 (**B**), HD-90R virus (**C**), HD-160A virus (**D**), HD-222Q virus (**E**), HD-227R virus (**F**), HD-244H virus (**G**) and HD-266T virus (**H**). Direct viral binding to either 3′SLN-PAA-biotin or 6′SLN-PAA-biotin sialylglycopolymers was determined. Representative data from three independent binding experiments are shown.

As shown in Figure 1, the wild-type HD/05 virus showing complete α-2,3 SA specificity and the 2009 pandemic H1N1 virus A/California/04/2009 (CA/09) binding selectively to α-2,6 SA were used as controls. The single amino acid substitutions K90R, K222Q, S227R, N244H and A266T in HA did not obviously affect the viral binding specificity to α-2,3 receptors at all, as compared with HD/05 virus. However, only the T160A mutant acquired affinity to α-2,6 receptors yet retained affinity to α-2,3 receptors, partially coincident with our previous publication indicating that this T160A-induced deglycosylation at site 158N contributed to the recognition of the human-type receptors as evidenced by hemagglutinin assay still with substantial agglutination to the α-2,3 sialidase pretreated goose red blood cells [23].

An experiment was conducted in guinea pigs to further investigate whether the T160A substitution of HA affected the replication of the H5N1 virus. Groups of four serologically H5-negative female animals were initially anesthetized with pentobarbital sodium (50 mg/kg), and inoculated intranasally with 10^6^ EID_50_ of the tested viruses in a volume of 300 µL (150 µL per nostril). Two animals from each group were humanely euthanized on day 3 post inoculation (pi) for virus titration from tissue samples of nasal wash, trachea, lung, kidney, spleen and brain. The remaining two animals were observed for signs of disease and death during a two-week period.

From Table 2, we found that when the 160A mutation was introduced in the HA of HD/05 virus, the replication of the mutated virus (HD-160A) in vivo dramatically increased. The mutant virus could replicate in the nose, lung and trachea of the guinea pigs with the highest titer level of 4.5 ± 1.2 log_10_EID_50_/mL, whereas the wild type HD/05 viruses were just detected in the lungs. However, virus was not recovered from the brains, kidneys or spleens of any of the H5N1 inoculated animals. Although no obvious disease signs were observed during 2 weeks pi, seroconversion was detected in both of the animals challenged with the mutated HD-160A virus but not identified in the HD/05-inoculated group.

**Table 2:** Replication and contact transmission of HD-160A virus in guinea pigs

Virus	Replication in guinea pigs							Contact transmission in guinea pigs	
	Virus titers determined on day 3 post inoculation in						Seroconversion: positive/total	Seroconversion: positive/total (HI titers)	
	Nasal wash (log_10_EID_50_ ± SD /mL)	Organ (log_10_EID_50_ ± SD /g)							
		Lung	Trachea	Spleen	Kidney	Brain		Inoculated	Contact
HD-160A	4.5 ± 1.2	2.5 ± 0.2	0.8 ± 0.1	–^a^	–	–	2/2	3/3(40,80,40)	2/3(0,20,20)
HD/05	–	1.2 ± 0.2	–	–	–	–	0/2	0/3	0/3

Groups of four guinea pigs were challenged intranasally with 10^6^ EID_50_ of either test viruses in a volume of 300 μL (150 μL per nostril), of which two animals from each group were euthanized on day 3 pi. Tissue samples involving nasal wash, lung, trachea, spleen, kidney and brain were collected for virus titration in eggs. Sera from the remaining two animals were collected at the end of the two-week observation period, and pretreated overnight with the receptor-destroying enzyme of Vibrio cholerae.

^a^Virus was not recovered from the undiluted tissue sample.

For viral contact transmission studies, three animals per group were infected intranasally at the dose of 10^6^ EID_50_ and housed in a cage placed inside a negative-pressure isolator. Another three naive animals were put into the same cage 24 h later to act as the contact counterparts. Nasal washes were collected at 2-day intervals since day 2 pi (1 day post contact) and stored at −70 °C until use for virus titration in chicken embryonated in eggs. All exposed animals were checked for seroconversion at day 14 pi via determination of hemagglutinin inhibition (HI) antibodies. To prevent inadvertent mechanical transmission of virus from investigators, the contact guinea pigs were always treated first, and operating implements were changed between animals. These studies were performed under ambient conditions of 20–22 °C and 20–40% relative humidity. The airflow in the isolator was kept horizontal with a constant speed of 0.1 m/s.

**Figure 2:**
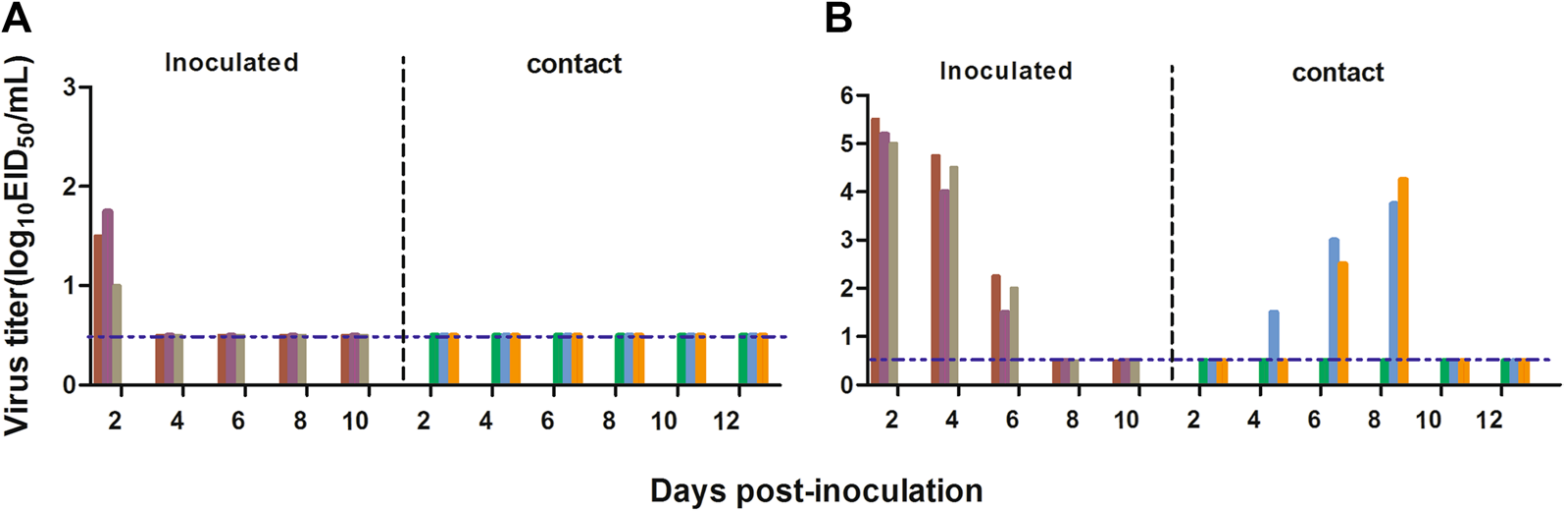
**Transmission of HD/05 virus and HD-160A virus in guinea pigs.** (**A**) HD/05 virus and (**B**) HD-160A virus. Groups of three guinea pigs were challenged intranasally with 10^6^ EID_50_ of either test viruses, and additional three contact guinea pigs were placed in the same cage 24 h after the inoculation. Nasal washes were collected every two days from all the animals since day 2 pi for detection of virus shedding. Each color bar indicates the viral titer from an individual animal. The dashed horizontal lines in the two panels represent the lower limit of detection.

As shown in Figure 2, the mutated HD-160A virus was detected in the nasal washes of all three inoculated guinea pigs from days 2 through 6 pi, and was also detected in two of the three contact animals between days 4 and 8 pi. In contrast, virus was not recovered from the nasal washes of any challenged guinea pigs after day 2 pi and of any contact animals in the group with the inoculum of wild-type HD/05 virus. Furthermore, seroconversion was also observed in three inoculated and two contact animals in the groups of the mutated HD-160A virus, but totally negative in the HD/05 virus group (Table 2).

## Discussion

Considering that the Asian lineage HPAI H5 subtype avian influenza viruses continuously expand both their geographical distribution and host range, whether they have gradually acquired efficient transmissibility between humans is of great concern. Although the natural isolates have still been deficient of such ability among people, two recent experimental studies report that reassortant H5N1 viruses with certain mutations in HA could gain the function of droplet transmission in a ferret model [24, 25]. According to submitted genome sequences in GenBank and GISAID, H5N1 clade 2.3.4 virus has caused the most human infections in China since 2005. In addition, the reassortant H5N6 virus within currently prevalent H5NX subclade 2.3.4.4 possessing dual affinity to α-2,3 and α-2,6 receptors, has also resulted in human death. Therefore, such above mentioned H5 viruses all pose a potential pandemic threat. In the present study, we compared the HA sequences of the previously described H5 viruses showing divergent receptor binding property to focus on potential adapted amino acid substitutions. And their effect on conferring viral affinity to α-2,6 receptors and transmission among mammalian models were then evaluated, based on the genetic backbone of a clade 2.3.4 virus preferentially binding to α-2,3 receptors. The results show that the single T160A substitution, which simultaneously led to the loss of glycosylation site at position 158N in HA dramatically enhanced the viral specificity for human-type receptors, and the HA mutant could spread efficiently in guinea pigs.

Glycosylation is an essential posttranslational modification for HA, actively involved in certain biological functions such as evasion of host immunity, HA cleavability and receptor binding [26]. The addition or elimination of the glycosylation site in close proximity to the RBD has been shown to modulate receptor binding specificity, antigenicity, and pathogenicity of influenza viruses of different HA subtypes and lineages [23, 27–29]. Furthermore, lack of the 158N glycosylation site in HA was found to be crucial for the transmissibility of H5N1 virus in mammals. It was demonstrated that the additive glycosylation at 158N due to an A160T mutation on a clade 2.2 virus, abolished the viral ability to bind to the α-2,6 SA and transmission in guinea pigs [30]. Moreover, reassortant H5N1 viruses of clade 1 and 2.1.3 HA with N158D-induced deglycosylation were capable of airborne transmission in a ferret model [24, 25]. However, whether this variation of the glycosylation pattern still works in the HA backbone of clade 2.3.4 influenza viruses remains unclear.

Here, we verified that the T160A mutation enabled H5N1 clade 2.3.4 virus to not only acquire binding affinity for human-type glycans but also transmit among guinea pigs. Although α-2,6 receptor binding property does not necessarily imply successful human transmission requiring additional synergy, mutants with high avidity for both α-2,3 and α-2,6 receptors may be intermediates during the evolution of novel H5 reassortants that could infect both poultry and humans to increase the risk of an influenza pandemic. Therefore, continued routine surveillance of H5 variants possessing dual receptor affinity is indispensable as an early warning system, and the presence or absence of the 158N glycosylation site could serve as an important molecular marker for assessing pandemic potential of H5 subtype avian influenza isolates.

## Additional file

**Additional file 1.** **Cartoon representation of the HA structure of A/mallard/Huadong/S/2005.** The model was generated by automated homology modeling using SWISS-MODEL based on the template of PDB (ID: 4jul). The receptor-binding domain (RBD) constituting with amino acids 188-190, 134-138, 221-228, 98,153 and 183 of HA1 (H3 numbering), according to reference [9], were colored yellow for clarity. The six divergent amino acids discussed in the text were colored gray, of which simultaneously located in RBD were labeled orange. The six specific substitutions were shown as sticks in the magnified side pictures, respectively. ‘‘K’’ is lysine, ‘‘R’’ is arginine, ‘‘T’’ is threonine, “Q” isglutamine, ‘‘S’’ is serine, ‘‘N’’ is asparagine, ‘‘H’’ is histidine and ‘‘A’’ is alanine.

## Abbreviations

HA: hemagglutinin
HPAI: highly pathogenic avian influenza
SA: sialic acid
RBD: receptor-binding domain
NA: neuraminidase
GISAID: global initiative on sharing all influenza data
HD/05: A/mallard/Huadong/S/2005
CA/09: A/California/04/2009
SPF: specific-pathogen-free
HRP: horse radish peroxidase
EID_50_: 50% embryo infectious dose titer
pi: post inoculation
HI: hemagglutinin inhibition
