# Supplementary figures and images for: The T160A hemagglutinin substitution affects not only receptor binding property but also transmissibility of H5N1 clade 2.3.4 avian influenza virus in guinea pigs
Source: Vet Res. 2017 Feb 6;48:7. doi: 10.1186/s13567-017-0410-0 (PMC5294818; doi:10.1186/s13567-017-0410-0)

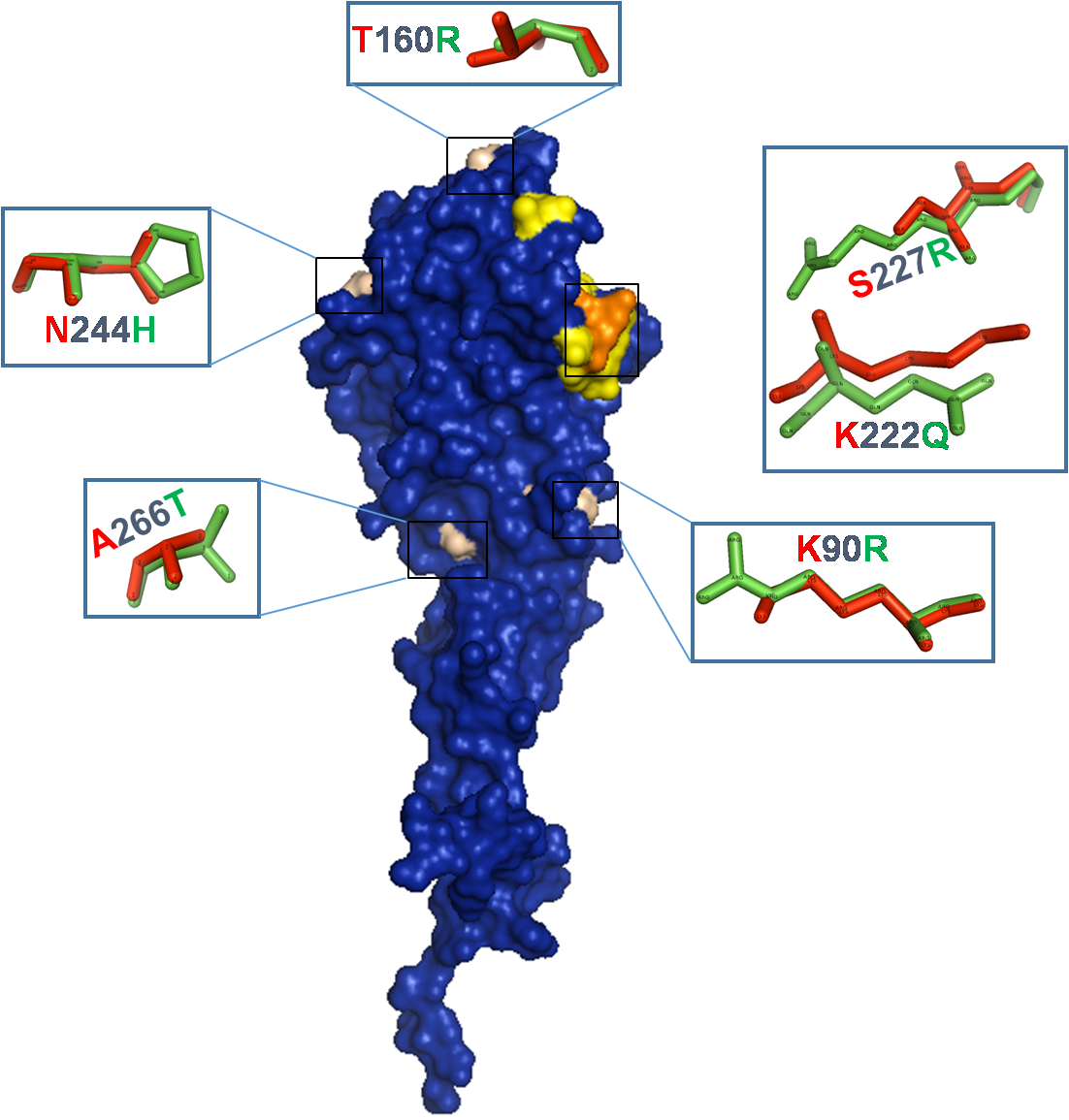

Supplement: Supplementary file 1 — Additional file 1. Cartoon representation of the HA structure of A/mallard/Huadong/S/2005. The model was generated by automated homology modeling using SWISS-MODEL based on the template of PDB (ID: 4jul). The receptor-binding domain (RBD) constituting with amino acids 188-190, 134-138, 221-228, 98,153 and 183 of HA1 (H3 numbering), according to reference [9], were colored yellow for clarity. The six divergent amino acids discussed in the text were colored gray, of which simultaneously located in RBD were labeled orange. The six specific substitutions were shown as sticks in the magnified side pictures, respectively. ‘‘K’’ is lysine, ‘‘R’’ is arginine, ‘‘T’’ is threonine, “Q” isglutamine, ‘‘S’’ is serine, ‘‘N’’ is asparagine, ‘‘H’’ is histidine and ‘‘A’’ is alanine. [file 13567_2017_410_MOESM1_ESM.docx]
